# Cohesin phosphorylation and mobility of SMC1 at ionizing radiation-induced DNA double-strand breaks in human cells

**DOI:** 10.1016/j.yexcr.2010.10.021

**Published:** 2011-02-01

**Authors:** Christina Bauerschmidt, Michael Woodcock, David L. Stevens, Mark A. Hill, Kai Rothkamm, Thomas Helleday

**Affiliations:** a CRUK/MRC Gray Institute for Radiation Oncology & Biology, University of Oxford, Old Road Campus Research Building, Off Roosevelt Drive, Headington, Oxford, OX3 7DQ, UK; b Health Protection Agency Centre for Radiation, Chemical and Environmental Hazards, Chilton, OX11 0RQ, UK; c Department of Genetics Microbiology and Toxicology, Stockholm University, S-106 91 Stockholm, Sweden

**Keywords:** ATM, SMC1, SMC3, Cohesin, Ionizing radiation, DNA repair

## Abstract

Cohesin, a hetero-tetrameric complex of SMC1, SMC3, Rad21 and Scc3, associates with chromatin after mitosis and holds sister chromatids together following DNA replication. Following DNA damage, cohesin accumulates at and promotes the repair of DNA double-strand breaks. In addition, phosphorylation of the SMC1/3 subunits contributes to DNA damage-induced cell cycle checkpoint regulation. The aim of this study was to determine the regulation and consequences of SMC1/3 phosphorylation as part of the cohesin complex. We show here that the ATM-dependent phosphorylation of SMC1 and SMC3 is mediated by H2AX, 53BP1 and MDC1. Depletion of RAD21 abolishes these phosphorylations, indicating that only the fully assembled complex is phosphorylated. Comparison of wild type SMC1 and SMC1S966A in fluorescence recovery after photo-bleaching experiments shows that phosphorylation of SMC1 is required for an increased mobility after DNA damage in G2-phase cells, suggesting that ATM-dependent phosphorylation facilitates mobilization of the cohesin complex after DNA damage.

## Introduction

Cohesin is a hetero-tetrameric protein complex consisting of SMC1, SMC3, Rad21 and either SA1 or SA2 in human cells [1,2]. The cohesin complex is involved in sister chromatid cohesion, DNA repair, and cell cycle checkpoint activation. It is loaded onto chromatin in the G1 phase with sister chromatid cohesion taking place in S which is crucial for accurate chromosome segregation in M [3].

Inverse fluorescence recovery after photo-bleaching (FRAP) was used to investigate how and when cohesin interacts with chromatin during the cell cycle [4]. Dynamically chromatin-bound cohesin was observed at constant levels throughout the entire interphase, but not on chromosomes in mitosis. Stably bound cohesin appears at the onset of S phase and persists on mitotic chromatin until it is cleaved by separase shortly before anaphase [4]. Cells in G2 show two populations of chromatin-bound cohesin. One population has a relatively short residence time of 8.4 ± 3.3 min and another which is bound so stably to chromatin that its residence time could not be determined over the observation time of 110 min [4,5].

The cohesin complex is known to accumulate at DNA double-strand breaks (DSB) in human cells [6] and in yeast where its loading is dependent on H2A phosphorylation [7,8]. In general, H2AX phosphorylation ( H2AX) is required for the concentration and stabilization of DNA repair proteins to damaged chromatin. Subsequently, other DNA damage mediators like MDC1 and 53BP1 are recruited to the damaged site, thus amplifying DNA damage signals [9,10].

SMC1 and SMC3 are phosphorylated by ATM [11–13] and cells show decreased survival and increased chromosomal aberrations after DNA damage [11]. It has been shown that MDC1 knockdown decreases the phosphorylation of SMC1 S996 in response to ionizing radiation (IR) [14] but does not abolish it.

Furthermore, cells defective in SMC1 and SMC3 phosphorylation show a defect in the S-phase checkpoint [12,15] and the G2/M checkpoint [16]. It is proposed that in contrast to cohesin's role in DNA repair, the checkpoint function of cohesin is independent of its ability to mediate cohesion [16]. However, the phosphorylation of SMC1 and SMC3 seems to be carried out only when these two subunits are part of the cohesin complex.

The aim of this study was to determine some of the requirements for SMC1/3 phosphorylation as well as its consequences on the spatiotemporal responses of these cohesin subunits following DNA damage. We show by siRNA depletion of RAD21 that only the fully assembled complex is phosphorylated and furthermore, that in humans ATM-dependent cohesin phosphorylation is mediated by H2AX, 53BP1 and MDC1, suggesting that only loaded cohesins close to DSBs are phosphorylated. We used fluorescence recovery after photo-bleaching (FRAP) to investigate the mobility of phosphorylated SMC1 in G2 phase cells. Our data suggest that ATM-dependent phosphorylation of cohesin is required for efficient mobilization of the complex after DNA damage.

## Materials and methods

### Antibodies

The following antibodies were used in this study: rabbit polyclonal against SMC1, SMC1pS966, SMC3, SMC3pS1083, MDC1 and H2AX (Bethyl Laboratories/Universal Biologicals, Cambridge, UK), anti-actin and anti-mouse/rabbit-HRP (Sigma, Dorset, UK), mouse monoclonal anti-Rad21 and anti-γH2AX (clone JBW301, Millipore, Dundee, UK), anti-CENP-F (ab5) and anti-53BP1 (Abcam, Cambridge, UK), anti-mouse AlexaFluor 488 (Invitrogen, Paisley, UK) and all other secondary antibodies for immunofluorescence (Jackson ImmunoResearch, Stratech, Suffolk, UK).

### Cell culture and synchronization

HeLa cells (Cancer Research UK, London, UK) were grown in Eagles minimum essential medium with 10% foetal calf serum, 2 mM l-glutamine, 100 U/ml penicillin, and 0.1 mg/ml streptomycin and cultured at 37 °C and 5% CO_2_. HeLa cells stably expressing EGFP-SMC1 wt or EGFP-SMC1S966A were generated by transfecting SMC1wt-pEGFP-N1 [5] or mutant SMC1-pEGFP-N1 using G-418 (Sigma, Dorset, UK) for selection.

Cell synchronization at the G1/S transition was achieved by a double thymidine block as previously described [17].

### Transfection

siRNA transfections were carried out using Oligofectamine (Invitrogen) according to the manufacturer's protocol. The siRNAs were either purchased from Dharmacon or from MWG Biotech (for details see Supplementary Table S1). All siRNAs were used at a final concentration of 100 nM. Cells were assayed 20–24 h after Rad21 siRNA transfection and 48 h after H2AX, 53BP1 and MDC1 siRNA transfection.

Plasmid transfections were carried out using polyethylenimine (PEI, Aldrich, Dorset, UK). For each well of a 6-well plate, 2.5 μg DNA and 10 μg PEI were mixed in 0.5 ml medium without FBS and antibiotics, incubated for 20 min at room temperature and slowly added to the cells. The medium was changed 4 h after transfection and cells were cultured with 700 μg/ml G-418 (Sigma, Dorset, UK) to establish stable cell lines. Clones were isolated and checked for their EGFP fluorescence. Clones with a stable nuclear expression of the EGFP-SMC1 were taken for subsequent FRAP experiments. Cells stably transfected with SMC1-EGFP have previously been shown to still form an intact cohesin complex containing the EGFP-SMC1 protein [5].

### Site-directed mutagenesis

A single point mutation (T2896G) in the cDNA sequence was generated in SMC1 wt to mutate Ser966 to Ala. Site-directed mutagenesis was carried out with the QuikChange® II XL Site-Directed Mutagenesis Kit (Stratagene/Agilent Technologies, Stockport, UK) according to the manufacturer's instructions. The following primer was used 5′AGG ACT CAG TGA GTG GTG CAC AGA GAA TTT CCA GT-3′.

### Irradiation

Conventional X-ray exposures were performed at room temperature with a 250 kV (constant potential) X-ray set (Pantak) with a compound filter of copper and aluminium (X-ray spectrum with a first half-value layer of 1.2 mm of copper) at a dose rate of ~ 1 Gy/min. Gamma-ray exposures were performed using a GSR D1 ^137^Cs γ-ray irradiator (Gamma-Service Medical GmbH, Leipzig, Germany) at a dose rate of ~ 1.7 Gy/min. Partial cell irradiation using partially shielded ultrasoft X-rays [18] was performed as described elsewhere [6].

### Immunofluorescence microscopy

Immunofluorescence was performed as described elsewhere [6]. Fluorescence images were captured using a Nikon Eclipse 90i fluorescence microscope equipped with cooled CCD camera and acquisition software.

### Fluorescence recovery after photo-bleaching

SMC1-EGFP cells were grown in T25 flasks and synchronized by double thymidine block [17]. Cells were placed into LabTek chambered cover glasses (Nunc/VWR, Lutterworth, UK) when released from the second thymidine block. Irradiation with a Cs-gamma irradiator was done 7 h after the second thymidine release. Cells were left to recover for 30 min in an incubator and then equilibrated in CO_2_ independent medium without phenol red (Invitrogen, Paisley, UK) supplemented with 10% FCS and antibiotics for another 30 min before imaging was started. Cells were kept at 37 °C on a heated stage insert (Zeiss, Germany) during imaging. Single cells were captured automatically with open pinhole using a Zeiss LSM710 microscope, Plan-Apochromat 63×/1.40 Oil DIC M27 objective. Nine iterations of photo-bleaching at 100% transmission of 488 nM laser were used for all FRAP experiments.

### Image processing and quantitative analysis

All cells that moved during the imaging process were excluded from analysis. To analyse FRAP data, fluorescence recovery was measured in user-defined cellular regions. The background-subtracted mean intensities were normalized to the initial fluorescence intensity distribution, thereby correcting for fluorescence decay due to post-bleach acquisition (Zen 2008 software, Zeiss, Germany).

The diagrams shown in Figs. 3D and E show only the mobile fraction of the EGFP-tagged protein. The programme used normalizes the data and takes the plateau reached during the observation period as 100% (Zen 2008, Zeiss, Germany). The bleaching introduced with the FRAP experiments was 75% +/− 2.2% for the un-irradiated and 79% +/− 3.4% for the irradiated cells. Residence times and immobile fractions were calculated using the same software. The significance of the difference in intensity curves between irradiated G2 cells stably transfected with SMC1 wt and SMC1S966A was tested by Student's t-test.

### Immuno-precipitation and Western blotting

Immuno-precipitations were carried out as described previously [17]. The immuno-precipitated proteins and possible co-IP partners were analyzed by Western blotting and were normalized to the signal intensity of the immuno-precipitated antibody band.

Cell pellets were lysed in ice-cold lysis buffer (1× TBS, 1% Triton X-100) containing protease inhibitors and 50 nM okadaic acid (both Sigma, Dorset, UK). Proteins were separated on NuPage Novex 3–8% Tris–Acetate or 12% Bis–Tris gels (Invitrogen, Paisley, UK) and then transferred onto a PVDF membrane according to the manufacturer's protocol (Invitrogen, Paisley, UK). Membranes were blocked in phosphate-buffered saline (PBS) containing 5% BSA. Primary and secondary antibodies were diluted in Tris-buffered saline/0.1% Tween20 (TBST) containing 2.5% BSA over night at 4 °C or for 1 h at room temperature. The proteins were visualised using Immobilon Western Chemiluminescent HRP Substrate (Millipore) and imaging was carried out using either a Kodak Image Station 4000MM system or Hyperfilm ECL films (GE Healthcare, Chalfont St Giles, UK). Quantification was performed using Kodak Molecular Imaging V4.0.1 software. The level of RNAi knockdown was determined using the level of actin as a standard.

### Flow cytometry

Cells were harvested and fixed with ice-cold methanol at 4 °C for a minimum of 30 min. For cell cycle analysis the fixative was removed by centrifugation at 250 *g* and the cells were resuspended in PBS containing propidium iodide (Sigma, Dorset, UK) at a final concentration of 10 μg/ml.

The samples were run on a Becton-Dickinson FACScan. Analyses were carried out using Flowjo (Treestar, Ashland OR 97520).

## Results

### SMC1 and SMC3 are phosphorylated when part of the cohesin complex

**Fig. 1:**
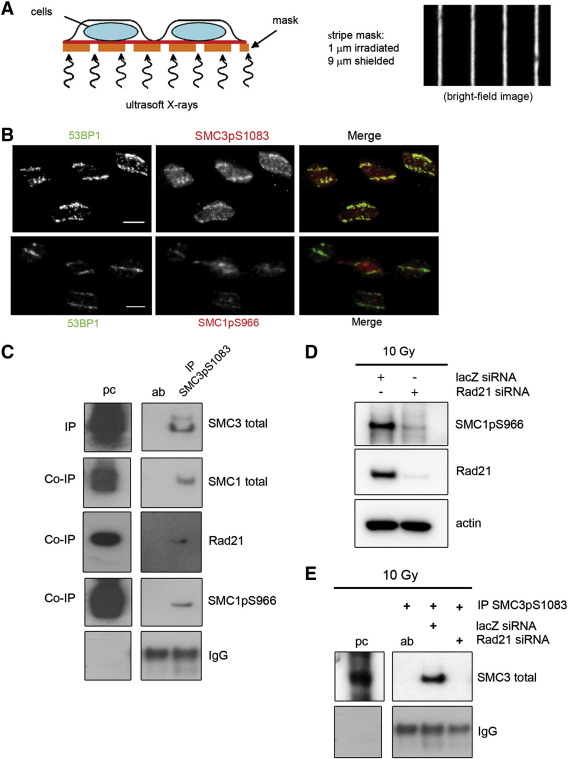
SMC1 and SMC3 are phosphorylated when part of the cohesin complex. (A) Defined ‘stripe’ patterns of localized DNA damage within the nucleus were achieved by using ultrasoft X-rays and a grid which contains 9 μm wide gold plates separated by a 1 μm wide open area as seen in the bright field microscopy image. (B) HeLa cells were grown on 0.9 μm thick Mylar for 24 h and then irradiated through the grid and the Mylar with ultrasoft X-rays. Cells were left for 1 h before fixation to allow DNA repair. Immunofluorescence microscopy for 53BP1 to detect areas of DNA damage and for SMC3pS1083 or SMC1pS966 was performed 1 h after ultrasoft X-ray irradiation. Scale bar 10 μm. (C–E) One representative blot is shown, two independent repeats. (C) 100 μg of un-irradiated or 10 Gy X-irradiated HeLa cell extracts was used for immuno-precipitation (IP) of SMC3pS1083. Precipitated proteins were analyzed by Western blotting. Panel shows (from top to bottom) precipitated SMC3 protein, co-precipitated total SMC1, Rad21 and SMC1pS966 and precipitated IgG antibody band. For lane ‘pc’ (positive control) 10 μg of X-irradiated extract (10 Gy) was loaded. Lane ‘ab’ shows the precipitated antibody. (D) HeLa cells were either transfected with non-specific siRNA (lacZ) or siRNA targeting Rad21. 20 μg of extract was loaded in each lane and Rad21 and SMC1pS966 levels were analyzed. Actin was used as loading control. (E) 100 μg of HeLa extracts treated with siRNAs for non-specific control (lacZ) or Rad21 was used for immuno-precipitation of SMC3pS1083. The precipitated SMC3 levels were analyzed on a Western blot. Antibody bands were used as loading control. For lane ‘pc’ 20 μg of X-irradiated extract (10 Gy) was loaded.

We used a partially shielded ultrasoft X-ray system (Fig. 1A) to determine the presence of phosphorylated SMC1 and SMC3 at sites of ionizing radiation (IR)-induced DNA damage. Immunofluorescence staining for DNA damage response proteins known to accumulate at damaged sites confirmed their recruitment to these localized damaged areas within the nucleus (Fig. S1). Similarly, SMC1pS966 and SMC3pS1083 were detected at these areas and co-localized with 53BP1 (Fig. 1B). The signals detected by the phospho-specific antibodies in X-irradiated samples were abolished by lambda phosphatase treatment, demonstrating that the antibodies specifically recognize the phosphorylated form of the proteins (Fig. S2). However, from these results we cannot exclude the possibility that other phosphorylated proteins are also picked up by these antibodies in immunofluorescence experiments.

To address whether phosphorylated SMC1 and SMC3 are part of the cohesin complex, we immuno-precipitated SMC3pS1083 from un- or irradiated HeLa cell extracts (Fig. 1C). SMC3pS1083 co-precipitated SMC1pS966, SMC1 and Rad21 (Fig. 1C) which indicates that the phosphorylated SMC subunits are part of the cohesin complex.

To test whether the cohesin complex had to be assembled to enable efficient SMC phosphorylation, siRNA was used to deplete the Rad21 subunit of cohesin (Fig. 1D). Surprisingly, we were unable to detect SMC1pS966 (Fig. 1D) and SMC3pS1083 (Fig. 1E) in Rad21-depleted extracts. We conclude that the phosphorylation of SMC1 and SMC3 requires Rad21, suggesting that only functional cohesin complexes are phosphorylated in response to IR.

We wanted to know whether RAD21 depletion results in changes of the cell cycle distribution and therefore this could explain the strongly reduced levels of SMC1 and SMC3 phosphorylation.

A cell cycle analysis was performed where HeLa cells were transfected with Rad21 or control siRNA, fixed 24 h post transfection and stained for PI to analyse cell cycle distribution. Rad21 depleted cells have a marginally different cell cycle distribution with more cells in G1 and less in S and G2/M. This marginal difference is statistically significant as shown by a t-test. The observed difference in relative numbers means 5% more G1 cells (55.94% vs. 50.75%), 3% less S cells and 2% less G2/M cells in Rad21 depleted cells (Fig. S3). We do not think that this observed difference explains the strongly reduced levels of SMC1 and SMC3 phosphorylation.

To determine the spatial distribution of SMC phosphorylation in relation to the DNA damage marker γH2AX in different cell cycle phases, co-immunofluorescence microscopy was performed with centromer protein F (CENP-F) which is absent in G1, increasingly abundant during S and peaks in G2/M [19]. γH2AX and foci for both phospho-SMC subunits co-localized in CENP-F negative and positive cells (Fig. S4) indicating that SMC1 and SMC3 are phosphorylated throughout the cell cycle following X-irradiation. Together, these data suggest that, in addition to mediating the S-phase checkpoint and promoting DSB repair in late S/G2, these phosphorylations may have other functions in the cellular DNA damage response.

### SMC1/SMC3 phosphorylation requires MDC1, 53BP1 and H2AX

To test whether generation of SMC1pS966 and SMC3pS1083 is H2AX-dependent in human cells and at which step in the signalling cascade the cohesin phosphorylation takes place, we used siRNAs to deplete H2AX, MDC and 53BP1 (for details see Supplementary Table S1).

**Fig. 2:**
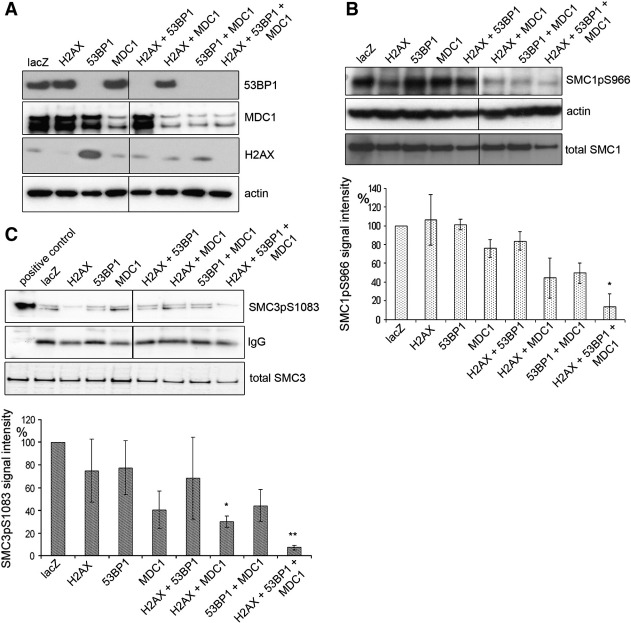
H2AX, 53BP1 and MDC1 promote phosphorylation of SMC1 and SMC3 following irradiation. (A) Western blot for H2AX, 53BP1 and MDC1 following siRNA depletion. HeLa cells were transfected with the indicated siRNAs (Supplementary Table S1), incubated for 48 h and X-irradiated with 10 Gy. Cells were harvested after 1 h to allow repair, 20 μg was loaded per lane and actin was used as loading control. (B) Western blot to detect SMC1pS966 and actin in 20 μg of extracts prepared in (A). SMC1pS966 signals were quantified using the Kodak Molecular Imaging software V4.0.1 and normalized to the actin signals (bar diagram). Total SMC1 signals are shown to ensure that SMC1 was present after each siRNA treatment. (C) Immuno-precipitation of SMC3pS1083 in 100 μg of extracts prepared in (A). pc = positive control. SMC3pS1083 signals were quantified as in (B) and normalized to the antibody bands (bar diagram). Error bars represent standard deviations of 2 independent experiments. The Student's t-test was used to compare lacZ siRNA-treated control to any other sample. P values between 0.01 and 0.05 are marked by ‘*’ and between 0.001 and 0.01 by ‘**’. Total SMC3 signals are detected on a Western blot run with the same extracts used for IP to show that SMC3 is present in the extracts.

HeLa cells were transfected with either a single siRNA or any combination of the three. Cells were irradiated 48 h following transfection and then left for 1 h to allow for DNA damage signalling and repair. The siRNA-treated extracts (Fig. 2A) were used to investigate the signal intensity of SMC1pS966 (Fig. 2B) and SMC3pS1083 (Fig. 2C). While 53BP1 depletion was very efficient, H2AX and MDC1 siRNAs led to only reduced protein levels. The residual protein will influence the observed phosphorylation of SMC1 and SMC3. Furthermore, 53BP1 depleted extracts show a highly reproducible increase in the H2AX signal.

However, SMC1pS966 and SMC3pS1081 were still present following any individual siRNA treatment and any combination of two of the siRNAs reduced the SMC3pS1083 signal to about 50% whereas the SMC1pS966 did not seem to be affected (Figs. 2B and C). Interestingly, when all three DNA damage mediators were targeted simultaneously, both phosphorylation signals were significantly reduced.

### SMC1 phosphorylation increases its mobility

FRAP experiments were performed to elucidate whether the phosphorylation of Ser966 has an influence on the mobility of SMC1. Stable cell lines were established expressing either wt EGFP-SMC1 or mutant EGFP-SMC1 where S966 was changed into an A. Relative expression levels of EGFP-SMC protein in these cells were assessed (Fig. S5) and the expression ratio of EGFP-SMC1wt to endogenous SMC1 (1.40) is similar to the ratio of EGFP-SMC1S966A to endogenous SMC1 (1.37).

These cells were synchronized by a double thymidine block, irradiated or sham-treated 7 h after release from the second thymidine arrest and incubated for 1 h before imaging was started.

**Fig. 3:**
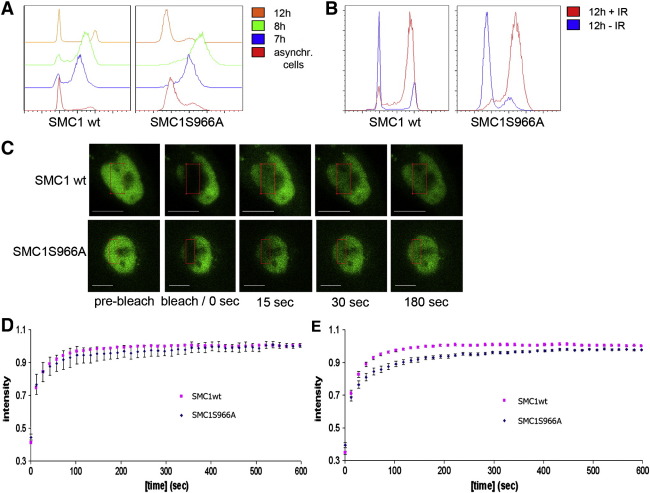
FRAP analysis of HeLa cells stably transfected with SMC1 wt or SMC1S966A. Cells were synchronized by double thymidine block, released for 7 h from the second thymidine block, either gamma-irradiated or left un-irradiated and analyzed by FRAP or flow cytometry. (A) Flow cytometry analysis of synchronized HeLa cells. Cells were fixed at the indicated time points and stained for propidium iodide. Asynchr. cells are logarithmically growing cells which serve as control for the set up of the flow cytometer. (B) Flow cytometry of 10 Gy-irradiated and un-irradiated cells 12 h after the release from the second thymidine block. Cells irradiated 7 h after the second thymidine release accumulated in G2 while un-irradiated cells moved into G1. (C) Dynamics of EGFP-SMC1 wt/S966A in 10 Gy-irradiated G2 cells. After pre-bleach scans, the fluorescence in the area indicated by the red rectangle was photo-bleached and the mean fluorescence was followed by time-lapse microscopy. Scale bars 10 μm. (D) Kinetics of EGFP-SMC1 wt and EGFP-SMC1S966A fluorescence recovery in un-irradiated G2 cells. Plotted is the mean intensity of the bleached region after normalisation to the pre-bleached images (mean ± SE; n (wt) = 48 cells, n (S966A) = 14 cells). (E) As in (D) but cells were γ-irradiated with 10 Gy, left for 2 h to allow repair and then analyzed by FRAP (mean ± SE; n (wt) = 26 cells, n (S966A) = 20 cells).

To determine the cell cycle stage during FRAP imaging, synchronized cells were fixed at indicated time points and stained with propidium iodide (Figs. 3A, B and S6). Irradiated cells showed a G2/M arrest compared to un-irradiated cells which moved through M into G1 (Fig. 3B). Therefore, G2 cells were imaged between 9 and 11 h after thymidine release and used for analysis (Fig. S6).

During FRAP imaging, one part of the nucleus in each cell was photo-bleached and the signal recovery was followed (Fig. 3C). Our experimental set up with an observation period of 14 min was designed to investigate the previously reported ‘mobile’ G2-phase population of cohesin with a residence time of 8 ± 3 min [4,5]. The diagrams shown in Figs. 3D and E show only the mobile fraction of the EGFP-tagged protein. The Zen programme (Zeiss, Germany) used for the analyses of the FRAP data normalizes the data and takes the plateau reached during the observation period as 100%. This explains the y-axis where the fluorescence recovery of the EGFP-tagged protein reaches 100% during the imaging period.

The bleaching introduced with the FRAP experiments was 75% +/− 2.2% for the un-irradiated and 79% +/− 3.4% for the irradiated cells. The calculated immobile fractions are 64% +/− 6.4% for the wt SMC1 and 51.2% +/− 8.8% for the mutant SMC1 (Fig. S7).

We analyzed the redistribution kinetics of irradiated and un-irradiated G2 cells by plotting recovery curves over time (Figs. 3D and E). The measured intensities show that the non-phosphorylatable SMC1S966A mutant has a significantly slower fluorescence recovery than wild type SMC1 (p ≤ 0.001) between 40 and 595 s in irradiated cells (Fig. 3E) while no significant difference was observed in un-irradiated G2 cells. The observed intensity curves indicate that wt SMC1 which can be phosphorylated on Ser966 after DNA damage is more mobile than the mutant SMC1S966A.

## Discussion

It has been shown that SMC1 and also SMC3 are phosphorylated by ATM [11–13]. SMC1/SMC3 phosphorylation is required not only for the DNA damage-induced intra-S-phase checkpoint [12,15,16] but also for the G2/M checkpoint, and that these functions are independent of cohesin's role in cohesion [16]. A function of cohesin in checkpoint control which is independent of its cohesion function supports our observation of a cell cycle-independent phosphorylation of the cohesin complex.

Using siRNA-mediated knockdown of Rad21, which is required to form a functional cohesin complex, we show that the phosphorylation of SMC1 and SMC3 requires fully assembled cohesin. It was shown previously that SMC1 phosphorylated on S957 can be immuno-precipitated with SMC3 indicating that it is part of the cohesin complex [15]. Furthermore, our findings are supported by Watrin and Peters who also show that SMC1/3 are phosphorylated as part of the cohesin complex [16]. Together, these data support the emerging picture that SMC1/3's phosphorylation-dependent functions outside their core role in sister chromatid cohesion may still depend on the fully assembled cohesin complex.

H2A phosphorylation is required for cohesin accumulation at DNA double-strand breaks in yeast [8]. In order to investigate whether H2AX and/or other mediators are required for efficient phosphorylation in human cells, we depleted H2AX, 53BP1 and MDC1 by siRNAs. One possible explanation for the observed gradual decrease in phosphorylated SMC1 and SMC3 could be the incomplete depletion of MDC1 and H2AX (Fig. 2A). However, these data support that H2AX, MDC1 and 53BP1 all modulate ATM kinase activity and/or ATM's interaction with SMC proteins. Our findings are supported by the earlier observation that MDC1 is required for efficient SMC1 phosphorylation [14]. As MDC1, H2AX and 53BP1 are involved in damage signalling in the vicinity of a DSB, the data imply that cohesin is phosphorylated in the chromatin region surrounding a DSB.

We used FRAP and measured the dynamic process of binding of cohesin after IR and its exchange over time where we focused on the influence of S966 on the mobility of SMC1. Cohesin consists of two populations, one fast pool with a residence time of 8.4 ± 3.3 min and a slow population with a residence time of more than 6 h in G2-phase cells [4,5]. Our experimental set up was designed to investigate the faster population, since phosphorylations occur rapidly in order to trigger an immediate response to DNA damage. We show that wild type SMC1 is more mobile than a non-phosphorylatable mutant (SMC1S966A). This provides the first evidence that cohesin's mobility is altered by this phosphorylation. It was shown previously that phosphorylation and acetylation of SMC3 are necessary for binding of cohesin to cohesin binding sites [20].

How SMC1 and 3 modifications influence cohesin binding mechanistically is currently not clear. We speculate that one functional consequence of SMC phosphorylation is to regulate the ATPase activity of the cohesin complex because the phosphorylation sites Ser966 on SMC1 and S1083 on SMC3 are close to the ATP hydrolysis sites in each SMC protein. Posttranslational modifications close to the ATP hydrolysis site might regulate this distinct step of the ATPase cycle. This is consistent with the observation that the ATPase function of the SMC1/3 heterodimer is essential for cohesin binding to chromatin and sister chromatid cohesion [21].

We speculate that ATP hydrolysis in the SA mutant is impaired and therefore movement of SMC1 after IR is slower than of wt SMC1. While SMC1S966A turned out to be slower in the beginning of the FRAP measurements the overall fraction of mobile protein has not changed (immobile fractions of wt and mutant SMC1 are similar). We speculate that the ATP hydrolysis is impaired and the mobility of the cohesin complex is altered because in the complexes with SA SMC1 and wt SMC3 only the SMC3 phosphorylation can influence the ATP hydrolysis.

One possible scenario could be the accumulation of cohesin at DNA DSBs due to the mobilized phospho-cohesin complex. Alternatively the observed enhanced mobility may suggest a release of phosphorylated cohesin subunits. This would result in more phospho-SMC1 floating freely in the nucleus which in turn would speed up fluorescence recovery. This may occur at sites of completed DSB repair, given that half of all DSBs are repaired within 1 h after irradiation.

This may only be one function of the phosphorylation sites of the cohesin complex and further investigation is needed to obtain a more complete picture of their functional relevance.
