# Supplementary material for: Cohesin phosphorylation and mobility of SMC1 at ionizing radiation-induced DNA double-strand breaks in human cells
Source: Exp Cell Res. 2011 Feb 1;317(3):330–7. doi: 10.1016/j.yexcr.2010.10.021 (PMC3087432; doi:10.1016/j.yexcr.2010.10.021)
Supplement: Supplementary file 1 — Supplementary materials. [file mmc1.pdf]

Figure S1

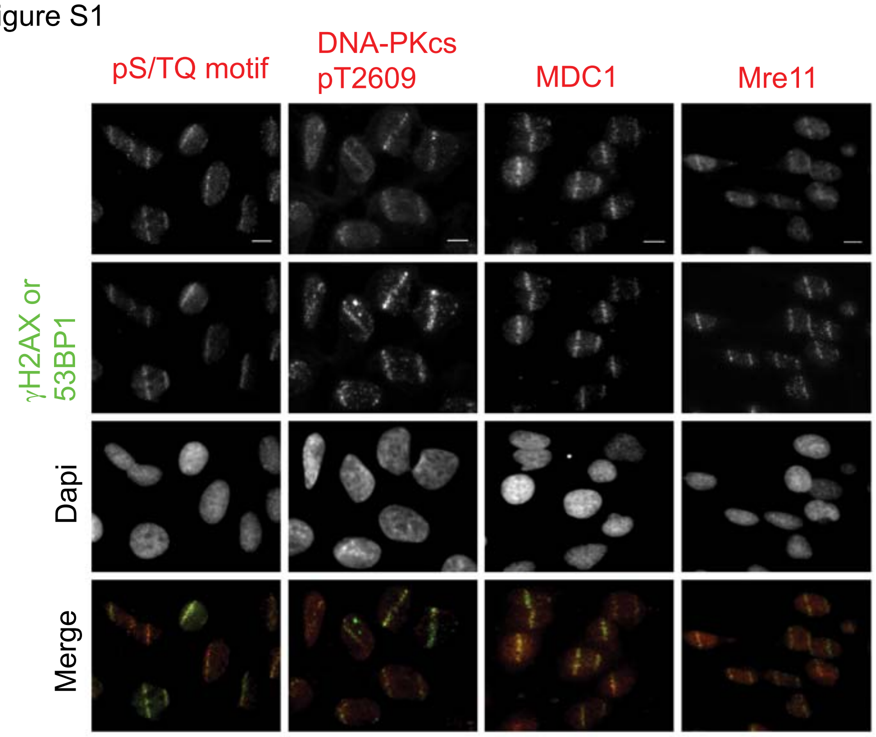

Figure S1.
Recruitment of DNA damage response proteins to subnuclear sites of DNA damage induced by partially shielded ultrasoft X-rays.
HeLa cells were grown on Mylar dishes for 24h and then irradiated using the ultrasoft X-ray grids described in legend to Figure 1. Either γH2AX or 53BP1 was used to visualize DNA damage.
Cells were co-stained for proteins known to accumulate at sites of DNA double strand breaks e.g. Phospho-(Ser/Thr) ATM/ATR substrate antibody (Cell Signaling), DNA-PK pT2609 (abcam), MDC1 (Universal Biologicals) and Mre11 (abcam): Scale bar 10 μm.

Figure S2

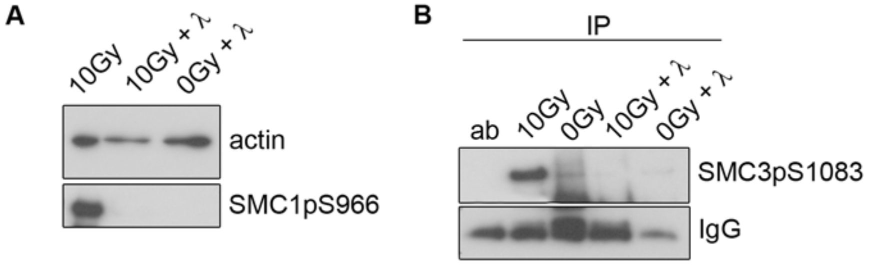

Figure S2.
Specificity of the anti-phospho-SMC1 and -SMC3 antibodies.
Lambda phosphatase was purchased from Upstate (Millipore, Dundee, UK). Phosphatase treatments were performed for 10 min at 37°C. The reactions were stopped by adding sodium bicarbonate to a final concentration of 1 M. **(A)** For Western blotting and SMC1pS966 detection 10 μg of either un-irradiated or X-irradiated extracts were incubated with 0.5 μg of lambda phosphatase (20.6 U/μg). Actin was used as loading control. **(B)** For immunoprecipitation and SMC3pS1083 detection 100 μg of either un-irradiated or X-irradiated extracts were incubated with 5 μg of lambda phosphatase (20.6 U/μl). Immunoprecipitated proteins were analyzed on a Western Blot. Antibody bands were used as loading control. Lane “ab” represents an immunoprecipitation of SMC3pS1083 antibody without extract incubation.

Figure S3

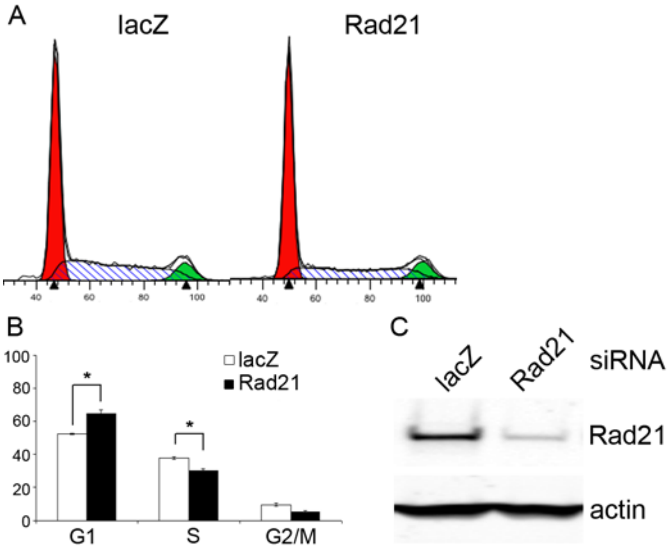

Figure S3.

Cell cycle analysis after Rad21 knockdown.

HeLa cells were transfected with control siRNA (lacZ) or Rad21 siRNA and irradiated with 10 Gy 24h after transfection. Cells were either fixed and stained with propidium iodide for flow cytometry (A and B) or harvested for Western blotting (C) one hour after irradiation.

Figure S4

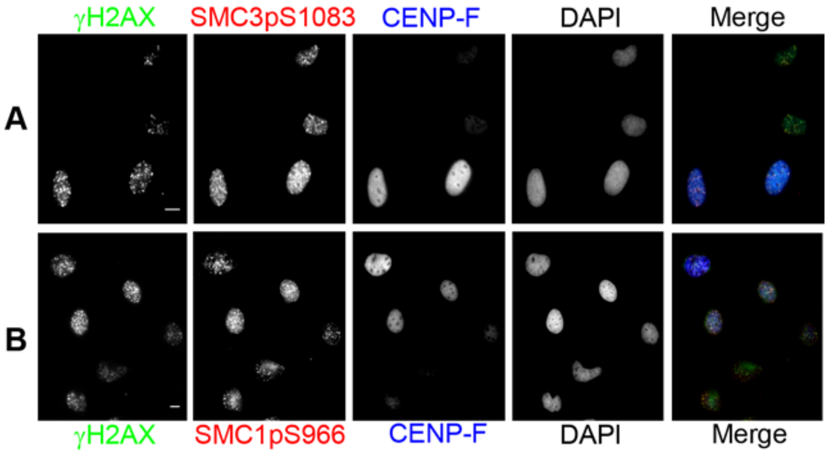

Figure S4.
SMC1 and SMC3 are phosphorylated throughout the cell cycle.
(A and B) HeLa cells were cultured on cover slips, irradiated with 1 Gy X-rays and fixed 1 h after irradiation. Cover slips were stained for γH2AX, CENP-F, either SMC3pS1083 or SMC1pS966 and DAPI. Scale bar 10 μm.

Figure S5

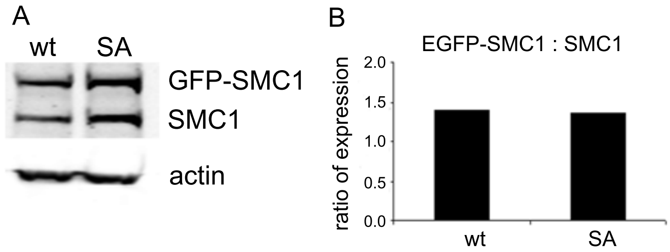

Figure S5: Relative expression level of EGFP-SMC1 wt and S966A.

The two clones with stable expression of EGFP-tagged wt or S966A SMC1 used for FRAP were analysed for their expression level of EGFP-SMC1 and endogenous SMC1 by Western blotting (A).

Here, secondary antibodies labelled with AlexaFluor 800 and the Odyssey system (LI-COR, Nebraska, USA) were used. Quantification was carried out using the quantification function implemented in the Odyssey imager (B).

Figure S6

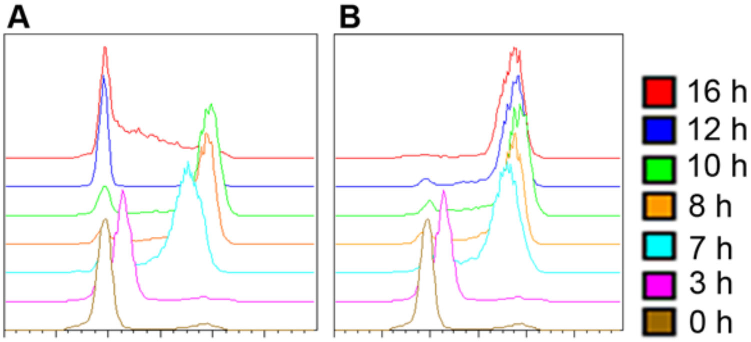

Figure S6.
Flow Cytometry analysis of HeLa cells stably transfected with SMC1 wt.
Cells were synchronised by double thymidine block, released for 7h from the second thymidine block, either not irradiated **(A)** or gamma-irradiated with 10 Gy **(B)**, fixed and stained with propidium iodide at the indicated time points and analyzed by flow cytometry.

Figure S7

A: row data

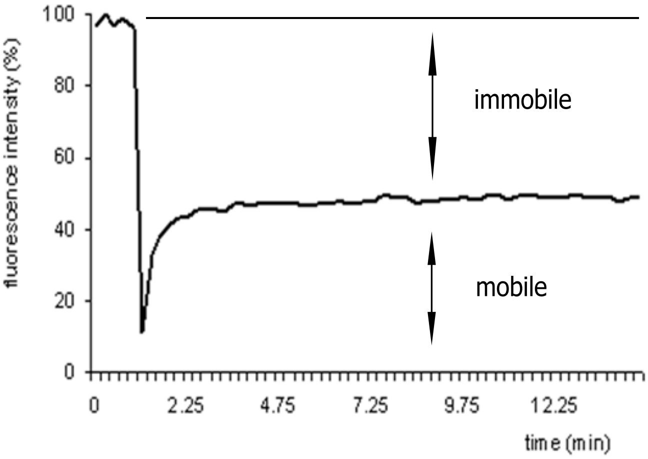

B: manually normalized data

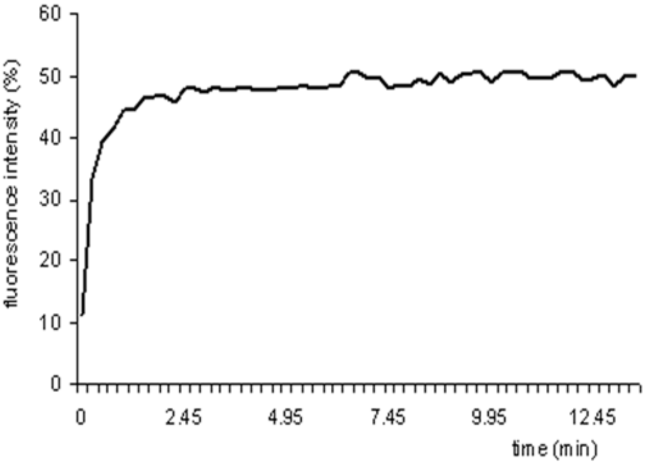

C: Zen programme normalized data

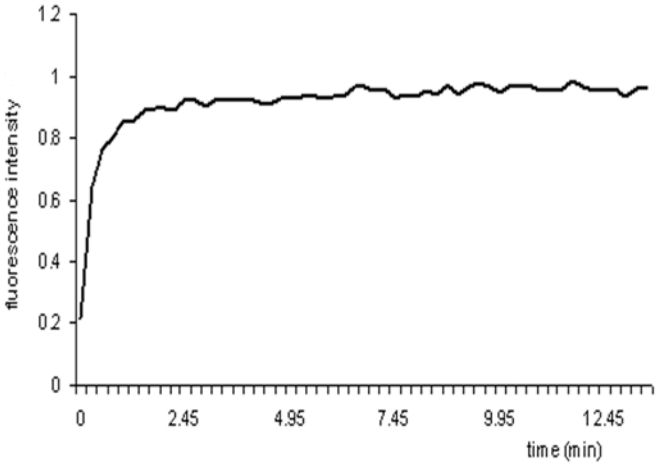

Figure S7: FRAP data analysis

One example of FRAP data analysis is shown. Data were aquired with a Zeiss confocal 710 microscope.
(A) Row data were taken into MS Excel and the measured background fluorescence was subtracted from the relative fluorescence of EGFP-SMC1S966A signals. Furthermore, all fluorescence values were converted into percentage of the initial pre-bleach fluorescence signals (100%).
(B) The first postbleach frame corresponds to t = 0 sec. The bleach introduced in this example reduced the fluorescence intensity of the EGFP-SMC1S966A to 11% of the initial fluorescence intensity. The immobile fraction in this example was calculated as 51%.
(C) The same data set was subjected to the analsysis done by the Zen programme (Zeiss, Germany). Here, the mobile fraction of the protein which reaches a plateau during the imaging time was taken as 100% and data was normalized accordingly.

Table S1: siRNAs used in Figures 1 and 2

siRNA target	sequence	Source / supplier
H2AX	5’-CAA CAA GAA GAC GCG AAU C-3’	(Lukas *et al.*, 2004) / Dharmacon
RAD21	5’-AUA CCU UCU UGC AGA CUG U-3’	(Losada *et al.*, 2005) / MWG Biotech
53BP1	5’-GAA CGA GGA GAC GGU AAU A-3’	(DiTullio *et al.*, 2002) / MWG Biotech
MDC1	5’-ACA GUU GUC CCC ACA GCC C-3’	(Stewart *et al.*, 2003) / MWG Biotech
LacZ	5’-CGU ACG CGG AAU ACU UCG A-3’	(Stewart *et al.*, 2003) / MWG Biotech

DiTullio RA, Jr., Mochan TA, Venere M, Bartkova J, Sehested M, Bartek J *et al* (2002). 53BP1 functions in an ATM-dependent checkpoint pathway that is constitutively activated in human cancer. *Nat Cell Biol* **4:** 998-1002.

Losada A, Yokochi T, Hirano T (2005). Functional contribution of Pds5 to cohesin-mediated cohesion in human cells and Xenopus egg extracts. *J Cell Sci* **118:** 2133-41.

Lukas C, Melander F, Stucki M, Falck J, Bekker-Jensen S, Goldberg M *et al* (2004). Mdc1 couples DNA double-strand break recognition by Nbs1 with its H2AX-dependent chromatin retention. *Embo J* **23:** 2674-83.

Stewart GS, Wang B, Bignell CR, Taylor AM, Elledge SJ (2003). MDC1 is a mediator of the mammalian DNA damage checkpoint. *Nature* **421:** 961-6.
